# How do digital access and remote consultations in primary care impact patients affected by domestic abuse?

**DOI:** 10.3399/BJGP.2025.0758

**Published:** 2026-08-01

**Authors:** Anna Dowrick, Sharon Dixon, Gene Feder, Lucy Downes, Estela Capelas Barbosa, Jasmina Panovska-Griffiths, Chris Griffiths, Eszter Szilassy

**Affiliations:** 1 Nuffield Department of Primary Care Health Sciences, University of Oxford, Oxford, UK; 2 Centre for Academic Primary Care, Population Health Sciences, Bristol Medical School, University of Bristol, Bristol, UK; 3 IRISi, Bristol, UK; 4 Pandemic Sciences Institute and the Queen’s College, University of Oxford, Oxford, UK

General practice can be a refuge for people experiencing abuse. For patients living under control and surveillance in a relationship, an interaction with primary care presents an opportunity to talk with a professional committed to values of autonomy and safety.^1^ The nature of the space in which this interaction takes place matters,^2^ as does how patients are identified, offered support, and directed towards specialist services.^3^ Safe and effective responses can have a profound impact on outcomes for patients and their families.^4^ This article discusses the impact of a shift towards digital access and remote consultations. It also considers ways in which general practice can adapt access routes and consultation modalities to better support patients affected by domestic abuse.

*“Beyond the everyday interactional challenges of patients connecting with professionals they trust, the broader enterprise is threatened by unanticipated work generated by the move to hybrid models of primary care access.”*

## The process of becoming a candidate for support

The collective and individual process of identifying a need for help and accessing services to meet that need has been characterised by Dixon-Woods as negotiating *candidacy*.^5^ Primary care is considered a *permeable* environment for identification of candidacy. In principle, any patient can appear at services and assert a need for support, which is followed by a negotiation with professionals about the best course of action. In reality, the ability to see oneself and be recognised by others as needing care is shaped by patterns of inequity and exclusion, which make requests for support easier for some patients than others.^6^

In the context of domestic abuse, it is challenging for patients to recognise and make visible that they need help. Abuse is hard to talk about and survivors fear judgement, having been made to feel worthless and undeserving of care through their abusive relationship.^7^ They might use a consultation to establish if a clinician is someone they can trust, starting with problems that they perceive are easier or more legitimate to talk about: headaches, chronic pains, tiredness. Patients need to believe that their clinician wants to hear what they have to say — they may build confidence in this through partial disclosure across multiple consultations. To overcome the barriers to disclosure, professionals have to create opportunities for conversation through establishing trust and offering safety and privacy. General practice clinicians are experienced at moving from a patient’s initial presentation towards other concerns and creating safe spaces for sensitive conversations, though remote consultations present new challenges.

## Navigating candidacy in a digital world

The shift to digital access and remote consultation means patients have more steps to take before they can have a private conversation with a clinician. Privacy is central to disclosure, but systems of digital access can make it challenging to guarantee. Dakin *et al* describe new work for patients in creating a ‘digital facsimile’, a digital version of themselves to convey the nature, seriousness, or trajectory of their problem when they seek help from general practice.^8^ It is on this digital facsimile that patients are judged and triaged, and on which the potential for in-person consultations is negotiated. Phone calls and digital interactions with primary care may be monitored by someone a patient is frightened of, which impacts what they can share. Privacy can be achieved in a remote consultation if patients are able to indicate a time for a call when it is safe for them to talk, though it can be preferable to offer a consultation room as a place of privacy. Patients who are known to general practice as needing support for domestic violence and abuse may be more likely to have support navigating towards privacy than others.

Rosen and Greenhalgh argue that patients with complex needs should get tailored triaging with a low threshold for transitioning to in-person consultations.^9^ For people who have never previously disclosed their troubles, the recursive loops of digital access and moving through different consultation modalities make it harder to be recognised as needing additional help. How patients not known to have complex needs are able to communicate this safely remains a challenge in digital access systems, with domestic abuse just one example.

Beyond the everyday interactional challenges of patients connecting with professionals they trust, the broader enterprise is threatened by unanticipated work generated by the move to hybrid models of primary care access. These systemic challenges have been well documented in this journal. Payne *et al* have exposed how changes in primary care access have had the consequence of dehumanising, compromising, and fragmenting care.^10^ Efforts to reorient practice towards team-working — internally within practices to share concerns about patients and externally with other agencies — may be stalled by the relational strain created through the effort of embedding challenging digital working routines.^11^ An important area for further work is exploring how the internal challenges of delivering remote services impact the care of and communication with vulnerable patients.

## How could we improve opportunities for safe disclosure?

Can we still create protected opportunities for disclosure? Can digital access systems work for patients who are not known as needing domestic abuse support, cannot easily say what they need from primary care, or perhaps don’t yet know? Primary care teams are not naïve to this challenge. GP practices have innovated strategies to better recognise patients remotely who might need additional support.^1^ Growing awareness of the prevalence, dynamics, and health impact of domestic abuse among primary care teams, largely through the evidence-based Identification and Referral to Improve Safety (IRIS) programme,^12^ supports teams to effectively identify and respond to patients experiencing abuse. Reception and administrative staff who triage patients have an increasingly important role in noticing subtle cues, such as hesitancy, when speaking on the phone, or changes in patient behaviour such as missed appointments. Appropriately and safely recording experiences of abuse in patient records has also become key in information sharing and in facilitating the continuity of a therapeutic relationship between the patient and a clinician, even when seeing the same professional is not possible. Discrete flags based on past experiences help to bypass complex triaging systems, regardless of the presenting condition, though require care as patient records might be viewed by other people. Third-party information from police, social services, and emergency departments facilitates the identification of patients who are at risk of violence and can enable priority access for face-to-face appointments. When patients are known as needing support, practice teams are able to work together to plan safe means of contacting them remotely.

## Conclusion

Digital access systems and remote consultations need to be made safer for patients affected by abuse. This can be done by providing opportunities for candidacy through easy, safe access systems, and prioritising privacy remotely or in person to enable opportunities for disclosure, in combination with advocacy and cross-service collaboration. This benefits all patients and is especially important for those whose vulnerability is not visible. Building stronger links with the specialist domestic abuse sector and primary care through clinical education and advocacy work remains key in supporting primary care teams in this delicate work, as does safely sharing information within primary care teams and across professions who witness different aspects of patients’ lives, such as police, social services, and secondary care services. This collaborative work bolsters the unique position primary care holds as a local source of support that anyone can seek help from, constituted of relationships that can foster hope.
